# Diagnostic performance of rapid antigen tests (RATs) for SARS-CoV-2 and their efficacy in monitoring the infectiousness of COVID-19 patients

**DOI:** 10.1038/s41598-021-02197-z

**Published:** 2021-11-24

**Authors:** John G. Routsias, Maria Mavrouli, Panagiota Tsoplou, Kyriaki Dioikitopoulou, Athanasios Tsakris

**Affiliations:** 1 Department of Microbiology, Medical School, National and Kapodistrian University of Athens, Athens, Greece; 2 GeneDiagnosis, Private Molecular Genetics Laboratory, Mihali Moraiti 93 & Andersen, Neo Psichiko, Athens, Greece

**Keywords:** Viral infection, Diagnostic markers

## Abstract

The most widely used test for the diagnosis of SARS-CoV-2 infection is a PCR test. PCR has very high sensitivity and is able to detect very low amounts of RNA. However, many individuals receiving a positive test result in a context of a PCR-based surveillance might be infected with SARS-CoV-2, but they are not contagious at the time of the test. The question arises regards if the cost effective, portable rapid antigen tests (RATs) have a better performance than PCR in identification of infectious individuals. In this direction, we examined the diagnostic performance of RATs from 14 different manufacturers in 400 clinical samples with known rRT-PCR cycles threshold (cT) and 50 control samples. Substantial variability was observed in the limit of detection (LOD) of different RATs (cT = 26.8–34.7). The fluorescence-based RAT exhibited a LOD of cT = 34.7. The use of the most effective RATs leads to true positive rates (sensitivities) of 99.1% and 90.9% for samples with cT ≤ 30 and cT ≤ 33, respectively, percentages that can guarantee a sensitivity high enough to identify contagious patients. RAT testing may also substantially reduce the quarantine period for infected individuals without compromising personal or public safety.

## Introduction

SARS-CoV-2 novel coronavirus emerged in China at the end of 2019 causing an ongoing pandemic. As of today, SARS-CoV-2 has affected the entire globe with over 100 million confirmed cases and 2.32 million deaths worldwide. Timely identification of SARS-CoV-2 infections is an urgent need for efficient epidemic control. Companies are continuously racing to develop the most sensitive detection test at all costs. However, the sensitivity of an assay neglects the context of how the test is being used. For example, a test used in effective surveillance regimens needs to deliver results timely to limit asymptomatic spread and should be inexpensive enough and easy to perform to allow frequent testing. The limit of its ability to precisely detect small concentrations of molecules (sensitivity) comes second. In certain cases, a very high sensitivity may be a pitfall, instead of a benefit. Thus, given that there is a long tail of RNA positivity after COVID-19 transmissible stage, the use of a high analytical sensitivity test detecting RNA has the drawback that many, if not most, people identified as positive are no longer infectious at the time of diagnosis. In this context a study conducted by the New York Times unveiled that in Massachusetts and New York, more than 50% of infections identified by PCR-based surveillance had PCR cycle threshold values (cTs) in the mid-to-upper 30s, indicating low viral RNA counts^1,2^. These results suggest that most patients (with minuscule viral loads) receive positive COVID-19 diagnoses after the infectious period has gone by, leading to unnecessary quarantining and contact tracing efforts.

Previous studies have indicated no recovery of infectious SARS-CoV-2 virus from specimens that produced cT values higher than 30^3–5^. Similarly, others found that patients with cT above 33–34 are not contagious and thus can be discharged from hospital care or strict confinement for non-hospitalized patients^6^. In addition, studies that examined SARS-CoV-2 on surfaces and air contamination, in an acute healthcare setting, also demonstrated that a PCR cT > 30 value indicates that the virus is not culturable^7^ and concluded that a cT value higher than 33 has no epidemiological relevance^8^.

The goal is to identify those who are currently transmitting the virus, meaning that we need tests that enable regimens to capture most infections while they are still infectious. Rapid Antigen tests (RATs), due to their high limit of detection, mostly identify patients that are actually contagious. Recently, Pekosz et al. demonstrated that RATs have a higher positive predictive value (90%) than rt-PCR (70%) when compared to culture positive results^9^. However, investigators, question the sensitivity of the RATs, their qualitative readout, their manufacturing quality and their discrepancies in diagnostic performance^10^. Our study aimed to answer these questions. In this regard, we examined the diagnostic performance of RATs from 14 different manufacturers using 400 clinical samples with known rRT-PCR cTs and 50 control samples.

## Methods

### Clinical samples and laboratory testing for SARS-CoV-2

This study was conducted in accordance with relevant official guidelines and regulations and approved by the Bioethics Committee of School of Medicine of the National and Kapodistrian University of Athens (ML-308-262021). All the samples were collected as part of routine diagnostic tests after written informed consent from all participants or, if participants were under 18, from a parent and/or legal guardian. Patients were all non-hospitalized who came voluntarily to our laboratories for COVID-19 testing, since they were symptomatic or close contacts of confirmed COVID-19 positive cases. Specimens were collected from both nostrils using the same swab by the same physician. The samples were transported in less than 15 min to the core laboratory at 2–8 °C according to the official national guidelines. The swab-derived eluates for which PCR assays gave a positive result were subsequently tested using different RATs. RT-PCR detection of SARS-CoV-2 was performed at the Genediagnosis (genetic diagnostic laboratory) using LiliF™ COVID-19 Multi Real-time RT-PCR Kit (INtRON Biotechnology Inc, IVD kit) that detects RdRP, E gene, N gene and as internal control the RNaseP gene. For the RNA extraction, the NX-48S, viral NA, CE IVD kit was used, on automated extraction system Nextractor^®^ NX-48S, CE IVD (Genolution Inc. Korea). Concerning the analysis of the PCR products, a cT value was assigned to each PCR reaction. 50 SARS-CoV-2 negative samples obtained from healthy subjects were used as controls.

### RATs

We compared RATs by 14 suppliers (Table 1), performing 400 evaluations of rRT-PCR-positive samples and 50 evaluations of rRT-PCR negative samples, according to the manufacturer’s instructions. Each swab eluate was tested against 4–8 RATs of different manufacturers. On average 29 different samples were tested via each RAT. The band intensity was assessed by:

**Table 1:** Limit of detection and manufacturer’s characteristics of RATs. Intensity: LOD determined by digital scanning. Visual: LOD determined by visual inspection. *LFIA* lateral flow immunoassay, *VFIA* vertical flow immunoassay, *LFFIA* lateral flow fluorescence immunoassay.

	Limit of Detection (PCR cT)			Method	Antigen	Manufaturer’s		Manufaturer
	Intensity	Visual	Average			Sensitivity%	Specificity%	
Wantai LFFIA	35.3	34.1	34.7	Fluorescence LFFIA	Nucleocapsid protein	95.71	100	Wantai SARS-CoV-2, LFFIA (Wantai Biological Pharmacy Enterprise Co., Ltd., Beijing, China)
Healgen	33.6	33.5	33.6	Colloidal Gold LFIA	Nucleocapsid protein	96.72	99.22	Healgen Coronavirus Ag Rapid Test Cassette (Healgen Scientific, Houston, Texas, USA)
Biosynex	32.4	32.5	32.5	Colloidal Gold LFIA	Nucleocapsid protein	96	100	Biosynex COVID-19 Ag BSS (BIOSYNEX SWISS SA, Suisse)
Wantai LFIA	32.5	32.1	32.3	Colloidal Gold LFIA	Nucleocapsid antigen	93.44	100	Wantai SARS-CoV-2 Antigen Rapid Test (Colloidal Gold) (WANTAI Biological Pharmacy Enterprise Co., Ltd., Beijing, China)
AMP	31.3	30.7	31	Colloidal Gold LFIA	Nucleocapsid antigen	97.3	100	AMP Rapid Test SARS-CoV-2 Ag (AMEDA Labordiagnostik GmbH, Graz, Austria)
LEPU	29.7	31	30.4	Colloidal Gold LFIA	Nucleocapsid antigen	92	99.26	LEPU SARS-CoV-2 Antigen Rapid Test Kit (Lepu Medical Technology Co., Ltd., Beijing,P.R. China)
Viva	30.2	29.5	29.9	Colloidal Gold LFIA	Nucleocapsid antigen	82.86	100	VivaDiag™ SARS-CoV-2 Ag Rapid Test (VivaChek Biotech Co., Ltd., Hangzhou, China)
Roche/SD-biosensor	30.1	29.3	29.7	Colloidal Gold LFIA	Nucleocapsid antigen	96.52	99.68	Roche/SD-biosensor Standard™ COVID-19 Ag (SD Biosensor Inc., Republic of Korea, distributed by Roche Diagnostics)
Hotgen	28.8	28.8	28.8	Colloidal Gold LFIA	N/A	96.62	99.76	Hotgen Novel Coronavirus 2019-nCoV Antigen Test (Hotgen Biotech Co., Ltd., Beijing P.R. China)
Salocor	28.6	28.2	28.4	Colloidal Gold LFIA	Nucleocapsid antigen	95	99.2	Salocor COVID-19 Antigen Rapid Test Cassette (Salofa Oy, Salo, Finland)
Dynamiker	27.6	27.7	27.7	Colloidal Gold LFIA	N/A	95	97.8	Dynamicer SARS-CoV-2 Ag COVID Rapid Test (Launch Diagnostics Ltd., Kent, England)
TestNow	27.5	27.2	27.4	Colloidal Gold LFIA	Nucleocapsid antigen	93.7	98.8	TestNOW®—COVID-19 Antigen (Affimedix Diagnostics, San Francisco CA, US)
CiTest	27.3	26.9	27.1	Colloidal Gold LFIA	Nucleocapsid & spike antigen	85	98.3	CITEST COVID-19 Antigen Rapid Test (Citest Diagnostics INC., Canada)
Coris	27.2	26.3	26.8	Colloidal Gold VFIA	Nucleocapsid antigen	60.1	99.2	Coris COVID-19 Ag Respi-Strip (Coris BioConcept, Belgium)

#### Visual assessment

RATs have been scored separately by two different investigators. Band intensity was graded as 0 (no visible band), 1 (faint line), 2 (faint band), 3 (weak band), 4 (clear band weaker than control) or 5 (clear band equal to or more intense than the positive control).

#### Digital scanning

Scanning was undertaken using UN-SCAN-IT Digitizer Software (Silk Scientific, Orem, UT, USA) for colorimetric quantification of bands. The intensity of the test line was expressed relatively to the intensity of the control line (as % percentage).

### Statistical analysis

The positive samples were compared using two by two contingency table. Agreement between RAT assay and RT-PCR was assessed using Cohen's k statistics. Pearson’s correlation test was performed to identify the relationship between the methods.

## Results

### Quantitative colorimetric read of LFIA tests is well correlated with their visual inspection

The intensity of each band obtained by digital scanning correlated perfectly with the score (0–5) obtained by the visual inspection and its classification according to the strength of the band (Pearson’s r = 0.903, p < 0.0001) (Supplementary Fig. S1).

### cT value directs the precise detection by RATs

**Figure 1:**
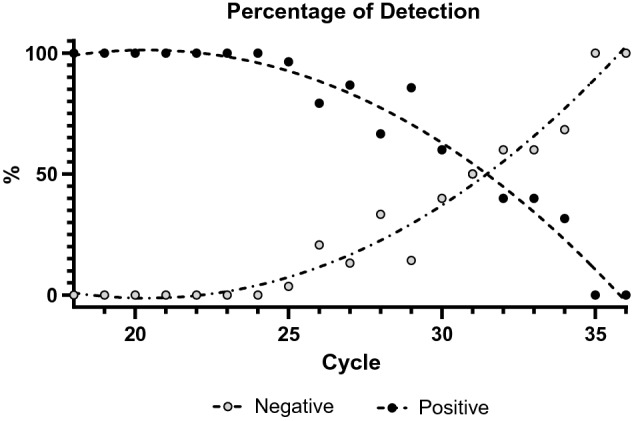
(**A**) The percentage of PCR positive samples that were found positive by RATs decreases as the PCR cT increases and the percentage of RAT-negative/PCR-positive samples is rising. 50% of samples are correctly identified as positive at cT = 31.5. (**B**) A significantly larger part of the RAT positive cases has cT values in the mid and lower range, while the highest cT values were more often observed in RAT negative cases.

The percentage of PCR positive samples that identified as positive by RATs drops as the PCR cT increases and concurrently the percentage of false negative samples increases (Fig. 1). A significantly larger share of the RAT positive cases had cT values in the mid and lower range, while the highest cT values were more often in RAT negative cases (Fig. 1).

### The intensity of the bands in RATs is reversibly correlated with cT. Substantial variability exists between the detection limits of individual RATs

**Figure 2:**
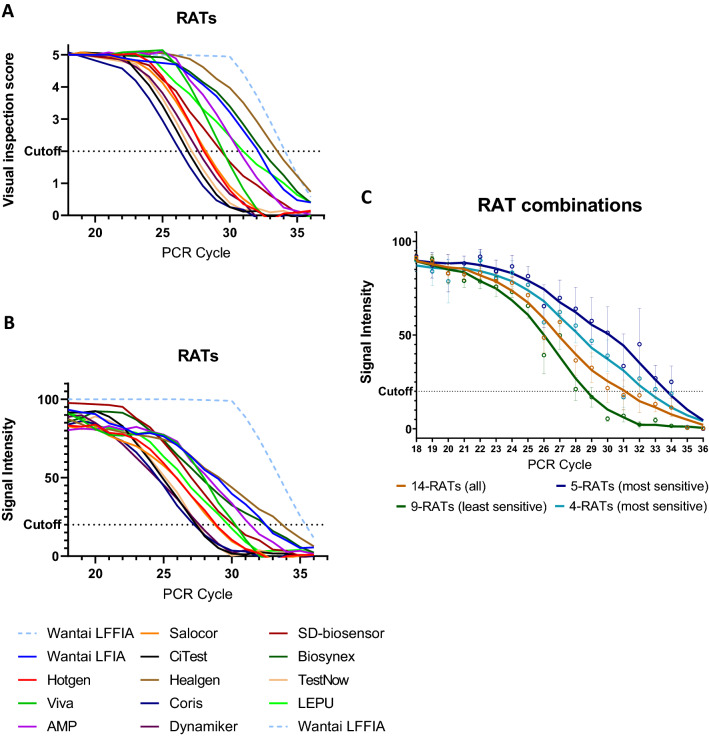
(**A**) A reverse correlation of the visual inspection score of the band with the PCR cT was found (Pearson’s r = − 0.704, p < 0.0001). The cutoff value of each RAT was determined as the average cT that produces a test band with at least a score of 2 in the optical observation (which can be surely visually observed). (**B**) A reverse correlation of the colorimetric intensity of the band with the PCR cT was found (Pearson’s r = − 0.733, p < 0.0001). The cutoff value of each RAT was determined as the average cT that produces a test band with an intensity of 20% compared to the control band. The detection limit varied between cT = 27.2 and cT = 33.6 amongst conventional individual LFIA/VFIA assays and was found at cT = 35.3 for the fluorescence LFFIA assay. Especially for the LFFIA assay, positive samples were considered to have an intensity of 100 and negative samples an intensity of 0, due to the lack of quantitative data. (**C**) RAT combinations. Overall, the detection limit of the 14 RATs tested was cT = 31.1. The best 5 (most sensitive) RATs, including the LFFIA assay, showed a detection limit of cT = 33.7 and excluding the fluorescence LFFIA assay (4 top) had a detection limit of ct = 32.5. On the other hand, the 9 less sensitive RATs showed a significantly lower detection limit of cT= 28.6.

For all 14 RATs obtained from different manufacturers there is a reverse correlation of the visual inspection score and the colorimetric intensity of the band with the PCR cT (Pearson r = − 0.704, p < 0.0001 and Pearson r = − 0.733, p < 0.0001, respectively) (Fig. 2). However, high differentiation and variability were observed regarding the performance of different RATs from different manufacturers. Although the vast majority of them succeeded in detecting the SARS-CoV-2 virus in samples with low or moderate cTs, only some of them succeeded it in higher cTs (Supplementary Figs. S2 and S3). In particular, the agreement of all 14 RATs with rRT-PCR at cTs < 27, was almost perfect (95.3%, k = 0.856), at cTs < 30 it was substantial (89.9%, k = 0.683), but at cTs = 31–35, it was low (58.9%, k = 0.139). The detection limit varied between cT = 26.8 and cT = 33.6 among conventional individual LFIA/VFIA assays and was cT = 34.7 for the fluorescence LFFIA assay (Fig. 2A,B and Table 1). Overall, the detection limit of the 14 RATs tested was cT = 31.1. The 5 best (most sensitive) RATs, including the LFFIA assay, exhibited a detection limit of cT = 33.7 and, excluding the LFFIA assay (4 best), had a detection limit of cT = 32.5. On the other hand, the 9 least sensitive RATs exhibited a significantly lower detection limit of cT = 28.6 (Fig. 2C).

### The sensitivity and specificity of RATs are dependent on the range of the cTs and the manufacturer

The overall sensitivity of all 14 RATs (regardless of cT values) was 74.3% (Table 2). However, the sensitivity depended on the cT and gradually decreased from 100 to 0% (Table 2). The specificity remained 100% in all cases. For cTs ≤ 30 and cTs ≤ 33, corresponding to the threshold values where SARS-CoV-2 is considered transmissible (according to different studies), the average sensitivity of all RATs was 88.2% and 80.0%, respectively. However, when we only took into account the 5 best RATS the sensitivity was found to be as high as 99.1% (for cT≤ 30) and 90.9% (for cT ≤ 33) (Table 2), while the agreement with rRT-PCR was perfect or almost perfect (99.4%, k = 0.986 for cT≤ 30 and 93.2%, k = 0.838 for cT ≤ 33).

**Table 2:** Sensitivity, specificity and agreement of RAT combinations at different cTs.

Tests	cT	Sensitivity	95% CI	Specificity (%)	95% CI	Agreement (%)	Cohen's k
14 RATs	All	74.25	69.67–78.47%	100	92.89–100%	77.11	0.391
14 RATs	< 20	100	92.45–100.00%	100	92.89–100%	100	1.000
14 RATs	21–25	98.18	93.59–99.78%	100	92.89–100%	98.75	0.971
14 RATs	26–30	76.26	68.31–83.06%	100	92.89–100%	82.54	0.630
14 RATs	31–35	37.5	27.82–47.97%	100	92.89–100%	58.9	0.291
14 RATs	> 35	0	0.00–19.51%	100	92.89–100%	74.63	–
14 RATs	≤ 30	88.18	83.90–91.68%	100	92.89–100%	89.94	0.688
14 RATs	≤ 33	80.00	75.52–83.98%	100	92.89–100%	82.41	0.491
5 top RATs	≤ 30	99.1	95.08–99.98%	100	92.89–100%	99.38	0.986
5 top RATs	≤ 33	90.85	85.12–94.91%	100	92.89–100%	93.10	0.830

Cohen's k: 0.01–0.20 slight agreement, 0.21–0.40 fair agreement, 0.41–0.60 moderate agreement, 0.61–0.80 substantial agreement, 0.81–1.00 almost perfect or perfect agreement.

### Detailed diagnostic performance of individual RATs

**Figure 3:**
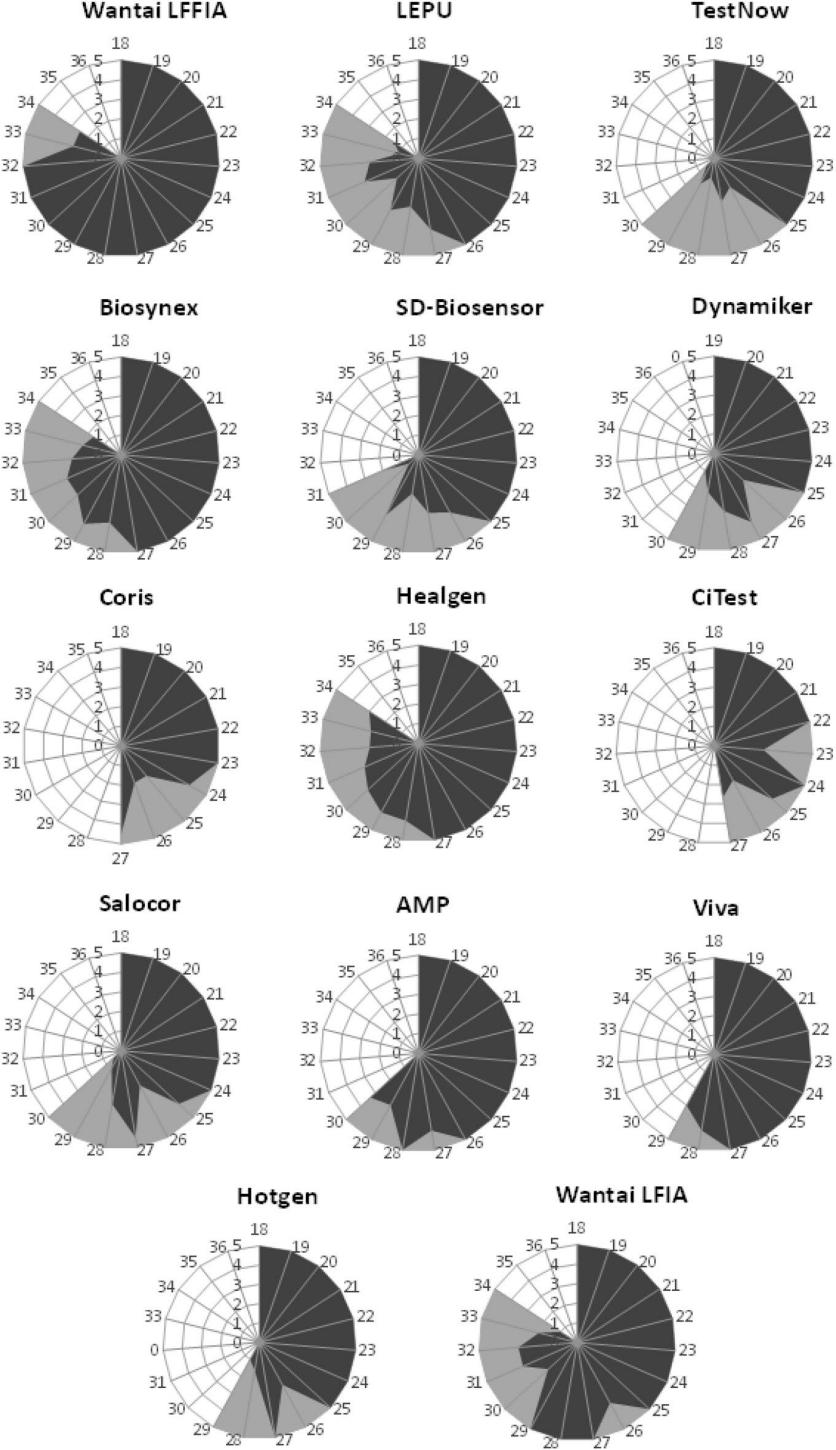
The diagnostic performance of each RAT is depicted as a spider graph. The length of each angular spoke (in dark grey) represents the average score (0–5) obtained by naked-eye visual inspection of the band for different samples of a designated cT. Different angles represent different cTs. The larger the area covered in blue, the strongest the test bands produced by this RAT. The second qualitative variable (in light gray) illustrates all the cTs of the samples that were successfully detected by this RAT and the area in gray defines the maximum sample’s cT that was found positive by this RAT.

As it is illustrated in Figs. 2 and 3 there is substantial variability in the diagnostic performance between different RATs. The LFFIA, an immunofluorescence-based assay read by a special instrument, has shown the best performance.

## Discussion

The goal of COVID-19 testing is to identify people who are currently transmitting the virus. The high sensitivity of the “gold standard” method for detection, the rRT-PCR, may be a pitfall since rRT-PCR can detect non-infectious, covered with antibodies, or dead virus particles and can remain positive for a prolonged period of time (even for months)^1,2,11–14^. This fact led the European Center for Diseases Control and Preventions (ECDC) to issue guidance for starting the 10-day isolation with a positive rRT-PCR test and discharge isolation of people with mild/moderate COVID-19 without a negative SARS-CoV-2 RT-PCR test. However, this guidance has two major problems, (a) at the beginning of isolation: if you do not take into consideration the cT value, many patients (with minor viral loads) receive positive COVID-19 diagnoses after their infectious period has passed, leading to potentially unnecessary quarantining and contact tracing efforts and (b) at the ending of the isolation without further testing: a small but significant percentage (up to 6%) of patients may still be contagious (especially on days 10–14)^12,15^ and the virus may also be culturable within the same time frame (10–20 days after onset of symptoms)^3,6^. Thus, the discharge of isolation without testing (at 10 days) has a “residual risk”. Additionally, the high sensitivity of the rRT-PCR assay may be related to true false-positive results. The false-positive results can occur either due to sample contamination (e.g. during the practice of “pooling of samples” that is applied in several cases for cost reduction) or a low-level contamination in the set of primers/probes used (regardless of the applied PCR chemistry)^16,17^.

In order to filter rRT-PCR results regarding infectivity several organizations used the threshold cycle limit of detection in PCR (cT)^2^. In this context the Health Protection Surveillance Centre of Ireland issued on 22.12.2020 Guidance on the management of weak positive (high cT value) PCR results, advising a patient with cT ≥ 35 to repeat the test after 2 days and in case the cT remains high (≥ 30), the person may generally be considered as a remotely acquired infection and non-infectious at the time of testing^18^. Moreover, the World Health Organization (WHO) issued a notice on 07.12.2020 informing labs that the cT cut-off should be manually adjusted to ensure that specimens with high cT values are not incorrectly assigned as “SARS-CoV-2 detected” due to background noise^19^. What if, another type of test could detect the virus with adequate (> 85%, as suggested by statistical models^13^, or > 80% as suggested by WHO^19^) but not with extremely high sensitivity that could augment background noise or detect samples with cTs > 34 as positive? Using this type of test would surely be beneficial for epidemic control. In this context, a recent report suggests that RAT testing more accurately reflects the presence of infectious virus in SARS-CoV-2-positive individuals, compared to the rt-PCR methodology^9^. However, the authors of the study question if their results will extrapolate to the other antigen tests due to variability in the limit of detection or other test characteristics^9,10^. Our study aimed to answer these questions and examine the characteristics of different RATs. In this regard we performed a head-to-head comparison of many RATs obtained from different manufacturers.

The intensity of the bands perfectly correlated with rRT-PCRs cTs (p < 0.0001). This finding could be useful for the development of cell phone applications allowing camera-reading of RATs, in a user-independent manner, with the potential of discrimination between marginal and strong positive tests and connection with real time COVID19 surveillance systems.

Significant variability was observed in the detection limit of different RATs (cT = 26.8–34.7). This partially explains the previously observed variability between individual RAT evaluations and is in concordance with the WHO’s warning on 11.09.2020, that many companies with low/moderate-quality products are entering the market with SARS-CoV-2 RATs^19^. The least accurate RATs can fail in unmasking a significant proportion of contagious patients. On the other hand, the use of the 5 most effective RATs can guarantee a sensitivity level high enough to identify contagious patients. In this regard, the overall detection limit of all RATs tested (cT = 31.1) can be significantly improved with the exclusive use of the 5 most sensitive RATs (cT = 33.7). Similarly, the selective use of the 5 best RATs increases the detection sensitivity from the acceptable rates of 88.2% and 80.0% (for samples with cT ≤ 30 and cT ≤ 33) to 99.1% and 90,9%, respectively, ratios ensuring correct identification of people currently transmitting virus.

The 100% specificity found in our study may represent the lower number of the evaluated normal samples (50 evaluations) than the number of the evaluated positive samples (400). The percentage of false positive samples in RAT testing in the literature is about 1% or less. Thus, with 50 evaluated normal samples it is possible not to meet this number. Since we used RATs of many different manufacturers and focused on the determination of the limit of detection (LOD) for each RAT, it was necessary to evaluate a large number of positive samples. The sensitivity found in our study, as categorized in different cT ranges, is in full concordance with previous studies that evaluated individual RATs with regard to the cT values of the samples^20,21^. If we do not consider the cT value, the sensitivity rates found (74.3%) are not directly comparable to these of other reports, since we have included only a few samples with very high cTs. In a systematic review and meta-analysis^22^, the average pooled sensitivity of RATs was found to be 56%. However, this sensitivity was calculated based on a PCR-based surveillance with data supporting that > 50% of infections identified have PCR cTs in the mid-to-upper 30 s, results linked to non-contagious individuals^1,2^. This is not the actual sensitivity of the test to detect people who are currently transmitting virus. Another study unveiled significant differentiation regarding RAT sensitivities between asymptomatic and symptomatic individuals (41.2% vs 80%), which were inversely proportional to the cT values in these groups (cT = 32.3 vs cT = 23.7)^23^. Virus was recovered from 32/39 (82.1%) of RAT(+)/PCR(+) samples, but only from 2/18 (11.1%) of RAT(−)/PCR(+) samples. Two more studies reported also that RATs are less effective in asymptomatic subjects than in symptomatic individuals^24,25^.

The detection limits that were found in our study for 14 RATs, cT = 31.1 for LFIA and cT = 34.7 for the LFFIA, correspond to nucleocapsid protein concentrations of 22 pg/ml and 3 pg/ml, respectively^26^, that are in complete agreement with their manufacturer’s specifications (20 pg/ml and 5 pg/ml, respectively).

In our study, both PCR and RAT tests were conducted using the same suspensions from the same nasopharyngeal swabs, and factors such as the operator, the tolerance of the patients and the sample volume, which are major variability factors, did not affect our results^27^. Additionally, the nucleic extraction and PCR amplification system used was the same in all cases and the visual assessment of all RATs was performed by the same investigators. A limitation of our study is that one cannot easily extrapolate and compare the cT cycles measured in our laboratory with the cT cycles measured by other laboratories due to differences regarding sample transport, RNA extraction, and performance of PCR assays with different primers and probes. Since, cT values can be affected by factors not related to the amount of virus in the specimen, CDC discourages the use of cT thresholds to measure viral load in individual cases^28^. Of course, the cT value is not the panacea for all ills, but at this point it is the only method we have to estimate the viral load, which is directly proportional to the virus transmissibility.

Our results suggest that the RATs, besides their low cost and ease of use, have the ability to identify contagious individuals. However, their analytic sensitivity, varies from manufacturer to manufacturer. The more sensitive RATs are able to detect the vast majority of contagious individuals and thus they can be beneficial in congregate settings, such as a long-term care facility or a correctional facility, workplace, or a school testing its students, faculty, and staff. Especially, for LFFIA, where the human eye is replaced by a more sensitive fluorescence reader, the detection limit was found at cT = 35. A previous study suggests that a similar fluorescence assay can detect samples of cT < 37 with a sensitivity as high as 91%^29^, which is more than sufficient for the surveillance and monitoring of transmissibility^18^.

RAT testing can also substantially reduce the quarantine period for COVID-19 cases without compromising personal or public safety^13^. Test-assisted quarantines could be proven safer and more cost-effective than 10/14-day quarantines. However, the effectiveness of a test-assisted quarantine strongly depends on test sensitivity requiring a rate higher than 85% to detect infectivity^13^. The 5 best RATs in our study are suitable for this purpose.

To gain control of the COVID-19 pandemic, the goal is to identify individuals currently transmitting virus and interrupt the transmission chains. The use of cost-effective, easy to use, rapid tests can accomplish this aim. However, due to discrepancies among the performance of different tests, a careful selection of RATs that meet the minimum cT detection limit criteria to confer high sensitivity is required.

## Supplementary Information

Supplementary Information.
