# Supplementary material for: Diagnostic performance of rapid antigen tests (RATs) for SARS-CoV-2 and their efficacy in monitoring the infectiousness of COVID-19 patients
Source: Sci Rep. 2021 Nov 24;11:22863. doi: 10.1038/s41598-021-02197-z (PMC8613285; doi:10.1038/s41598-021-02197-z)
Supplement: Supplementary file 1 — Supplementary Information. [file 41598_2021_2197_MOESM1_ESM.pdf]

Diagnostic performance of Rapid Antigen Tests (RATs) for SARS-Cov-2 and their efficacy in monitoring the infectiousness of COVID-19 patients

John G Routsias*, Maria Mavrouli*, Panagiota Tsoplou#, Kyriaki Dioikitopoulou# and Athanasios Tsakris*

*Department of Microbiology, Medical School, National and Kapodestrian University of Athens, Athens, Greece

#GeneDiagnosis, Private Molecular Genetics Laboratory, Mihali Moraiti 93 & Andersen, Neo Psichiko, Athens, Greece

Corresponding author:
John G. Routsias Tel.: +30 210 7462133; fax: +30 210 7462210. E-mail address: jroutsias@med.uoa.gr

Running title: Diagnostic performance of Rapid Antigen Tests

Key words: COVID-19, SARS-CoV-2, Rapid Antigen Tests, rRT-PCR

Word count: 2584 words

Legends to the supplementary figures

Figure S1

The relative intensity of each band obtained by digital scanning (as % of the difference in intensity to the control band) correlated perfectly with the score (0-5) obtained by the visual inspection of the line (Pearson's $r=0.903$, $p<0.0001$).

Figure S2

We observed substantial differentiation and variability regarding the performance of different RATs from different manufacturers. Both PCR and RAT tests were conducted using the same suspensions from the same nasopharyngeal swabs. Panels A-D depict the performance of different RATs for 4 nasopharyngeal swabs with cTs: 34,27,27 and 32, respectively. Although all the RATs succeeded in detecting the SARS-Cov-2 virus at ct=27 (panels B,C), only some of them achieved it in higher cTs (eg at cT=34, panel A and cT=32, panel D)

Figure S3

Average intensity of the bands obtained from the same RAT for samples of the same cT. The detailed colorimetric read of LFIA tests shows substantial variability in the performance of different RATs. For the LFFIA assay, positive samples were considered to have an intensity of 100 and negative samples an intensity of 0, due to the lack of quantitative data.

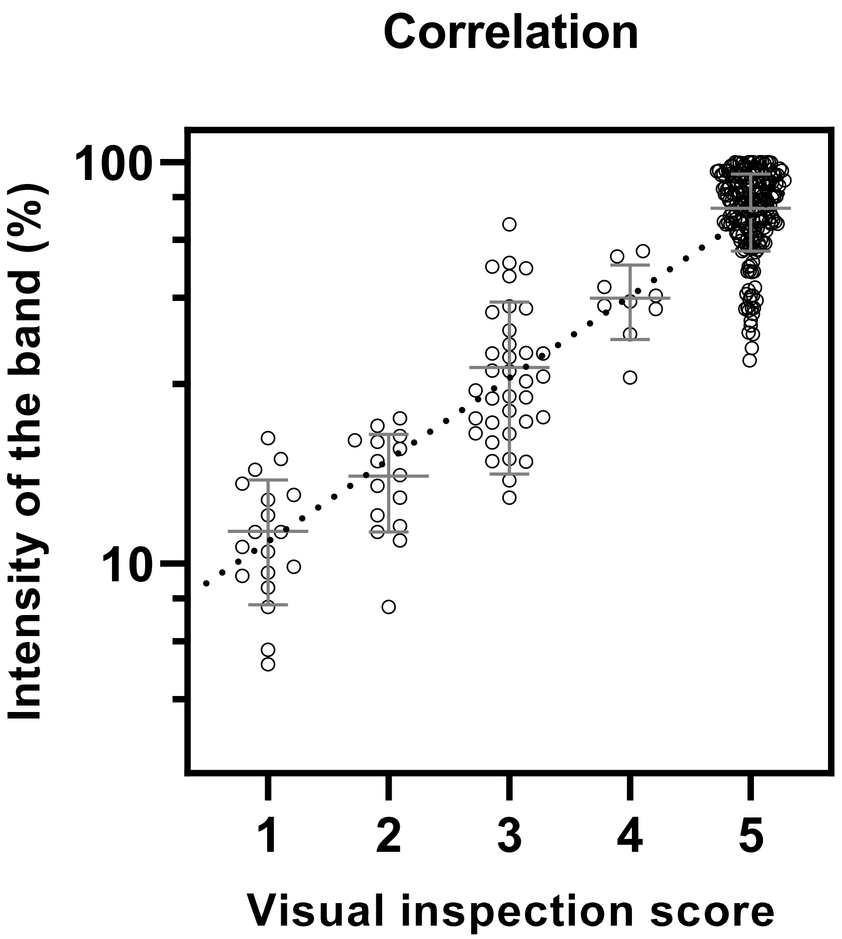

Supplementary Figure S1

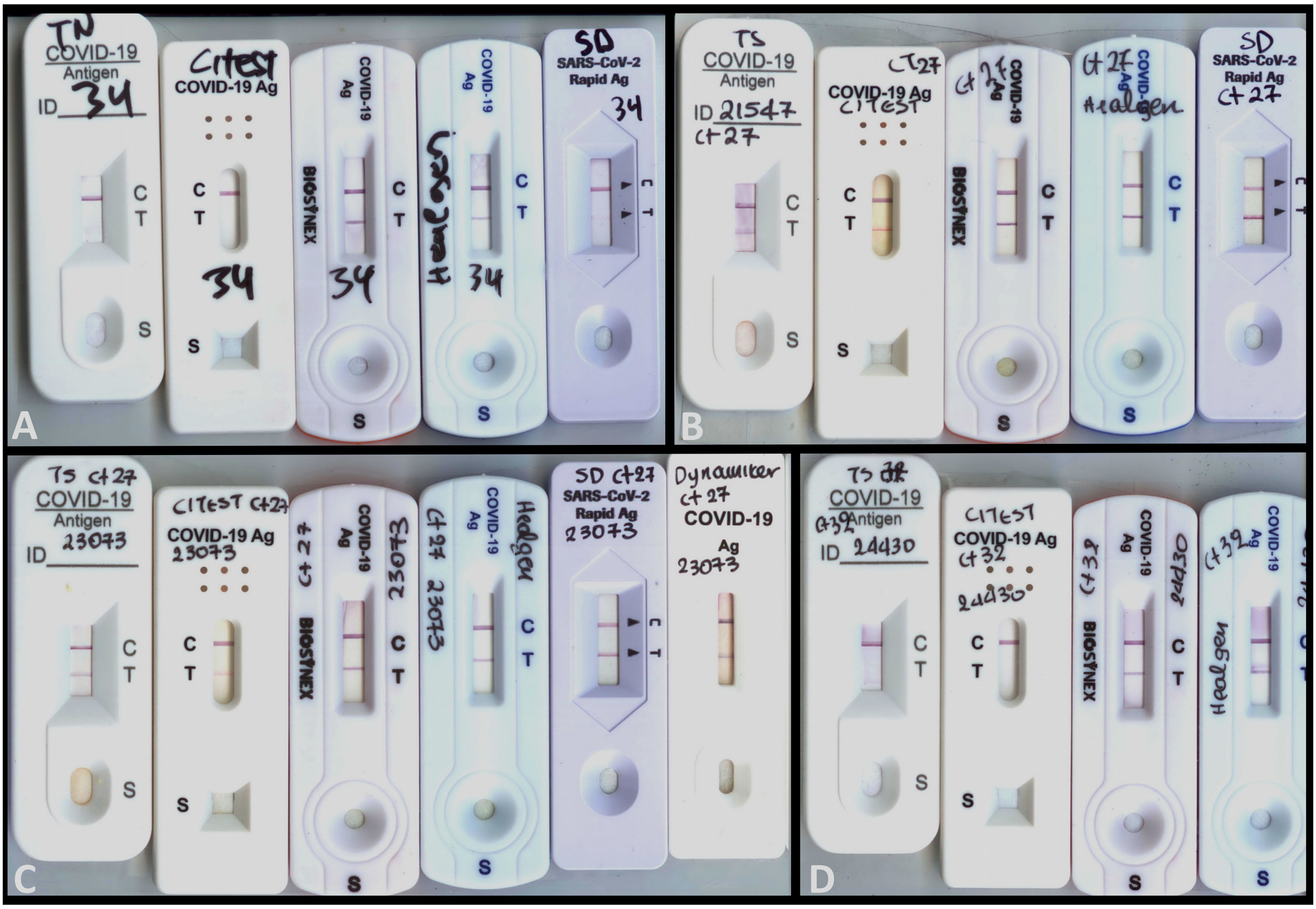

Supplementary Figure S2

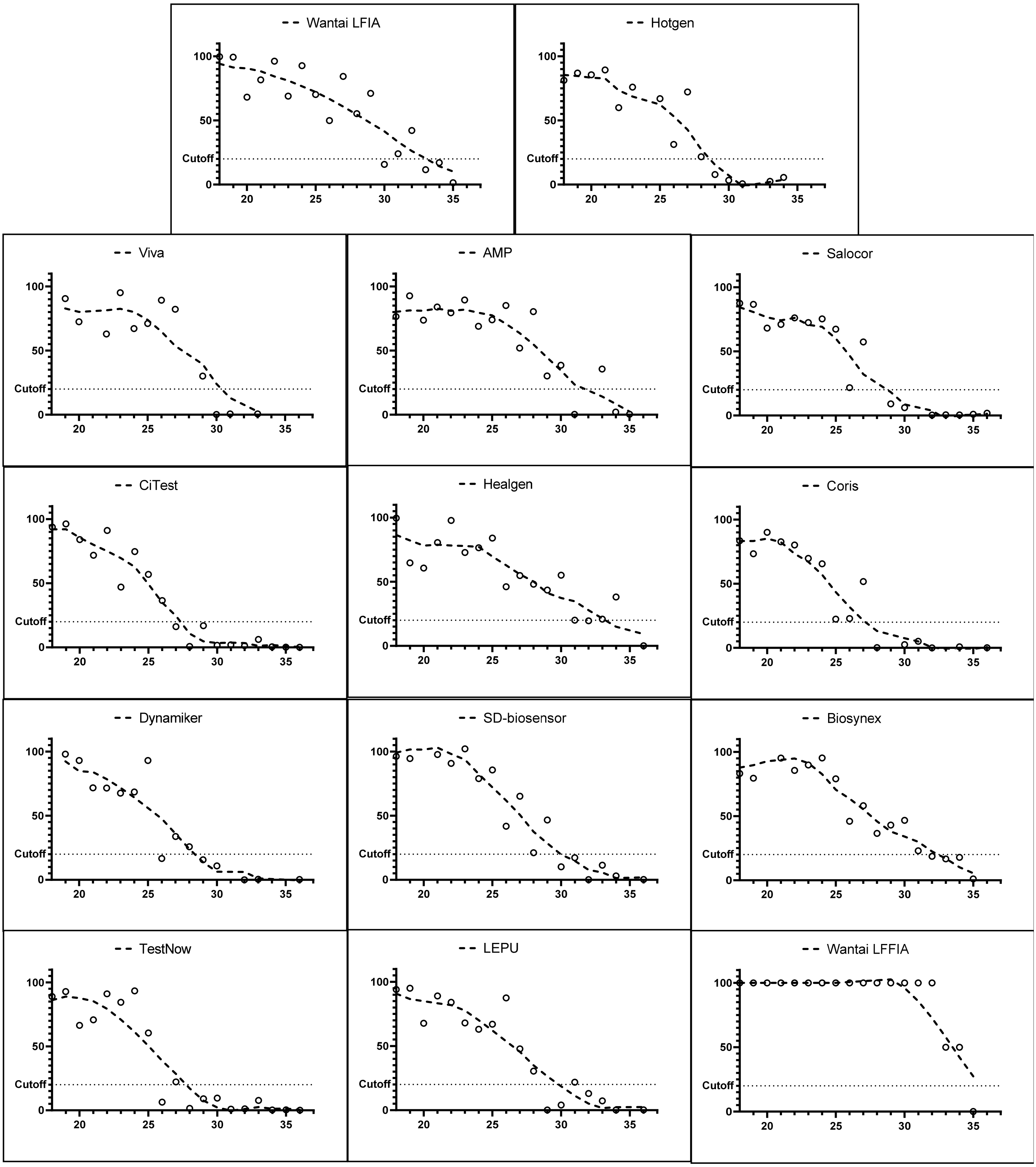

Supplementary Figure S3
